# Gut microbiota and autoimmune thyroid disease: a bidirectional Mendelian randomization study and mediation analysis

**DOI:** 10.3389/fmicb.2024.1443643

**Published:** 2024-09-16

**Authors:** Yiqiao Fang, Xinyue Zhang, Rui Huang, Jiaye Liu, Zhihui Li

**Affiliations:** ^1^Division of Thyroid Surgery, Department of General Surgery, Laboratory of Thyroid and Parathyroid Diseases, Frontiers Science Center for Disease-Related Molecular Network, West China Hospital, Sichuan University, Chengdu, China; ^2^Department of Respiratory and Critical Care Medicine, Frontiers Science Center for Disease-Related Molecular Network, Center of Precision Medicine, Precision Medicine Key Laboratory of Sichuan Province, West China Hospital, Sichuan University, Chengdu, China; ^3^Department of Nuclear Medicine, West China Hospital, Sichuan University, Chengdu, China

**Keywords:** autoimmune thyroid disease, gut microbiota, immune cell traits, Mendelian randomization analyses, mediation analysis

## Abstract

**Background:**

The gut microbiota (GM) plays a pivotal role in influencing various health outcomes, including immune-mediated conditions, but its potential association with autoimmune thyroid disease (AITD) remains underexplored. We aimed to investigate the potentially pathogenic or protective causal impacts of specific GM on two types of AITD, namely Graves’ disease and Hashimoto’s thyroiditis, and analyzed the mediating effect of 731 immune cell phenotypes.

**Methods:**

Leveraging pooled genome-wide association study (GWAS) data of 211 gut microbiota traits, 731 immune cell phenotypes, and two types of AITD (Hashimoto’s thyroiditis and Graves’ disease), we performed bidirectional Mendelian randomization (MR) analyses to explore the causal relationships between the GM and AITD. Subsequently, we employed a multivariable MR analysis to discover potential mediating immune cell traits. Additionally, sensitivity analyses were utilized to ensure the reliability of the outcomes.

**Results:**

Our analysis revealed that a total of 7 GM taxa were positively associated with AITD, and other 14 taxa showed a negative correlation with AITD. Furthermore, we identified several immune cell traits that mediated the effects of GM on AITD. Most notably, *Actinobacteria (p)* presented protective effects on Hashimoto’s thyroiditis via CCR2 on myeloid Dendritic Cell (5.0%), and *Bifidobacterium (g)* showed facilitating effects on Graves’ disease through CD39+ CD4+ T cell %CD4+ T cell (5.0%) and CD14 on CD33+ HLA DR+ CD14dim (12.2%).

**Conclusion:**

The current MR study provides evidence supporting the causal relationships between several specific GM taxa and AITD, and further identified potential mediating immunophenotypes.

## 1 Introduction

Autoimmune thyroid disease (AITD), mainly including Graves’ disease (GD) and Hashimoto’s thyroiditis (HT), affects 5% of the general population, making it one of the most prevalent autoimmune diseases (Antonelli et al., 2015; Lee et al., 2015). Despite the different clinical manifestations, GD and HT share similar immune-mediated mechanisms of disease, even alternating from one to the other (Lee et al., 2015). As already stated, the onset of AITD implicates a breakdown of immune tolerance towards the thyroid, through an autoimmune multifactorial process, involving environmental and endogenous factors in genetically susceptible individuals. The etiology of AITD is currently considered as a complex interplay of specific susceptibility genes and environmental exposures (Vargas-Uricoechea, 2023). However, current treatments aim to control hormone-related symptoms, and have been proven ineffective in correcting the dysregulated immunity in patients, making it essential to develop novel targeted prevention and treatment strategies.

Gut microbiota (GM) refers to all micro-organisms that inhabit the human gastrointestinal tract and are considered as the largest reservoir of microbes in the human body (Lynch and Pedersen, 2016). Due to the intricate and symbiotic relationship with the host, the GM is closely related to various aspects of human health, not just intestinal diseases (Valdes et al., 2018). From the embryology aspect, the thyroid and gut share a common embryological origin, explaining some morphological and functional similarities between the gut and thyroid follicular cells (Lahner et al., 2020). Previous evidence for possible GM involvement in the onset and progression of AITD was based solely on retrospective measures of bacterial antibodies in AITD patients (Strieder et al., 2003; Larizza et al., 2006; Bassi et al., 2010). Until now, there is no direct evidence that AITD and gut microbiota have a cause-effect relationship.

Mendelian randomization (MR), using genetic variants as instrumental variables (IVs), is a widely accepted method to control potential confounding factors, which can avoid reverse causation bias and allow more robust causal inferences between exposure and clinical outcomes (Sekula et al., 2016). Two-sample MR analysis utilizes single-nucleotide polymorphism (SNP)-exposure and SNP-outcome associations from independent genome-wide association study (GWAS) and combines them into a single causal estimate. As the number of GWASs on GM and diseases has increased rapidly, large-scale summary statistics have become more widely available, allowing for boosting studies on GM MR analysis with significantly improved statistical power (Li et al., 2022; Gagnon et al., 2023; Xu et al., 2023).

Although previous observational studies have emphasized the link between altered microbial diversity and AITD, but a causal relationship has not been established. The present study aims to elucidate the specific gut microflora that potentially contribute to the onset of AITD and assess their potential as novel targets for treatment. We employed bidirectional MR dissect the causal impact of GM on two types of AITD (GD and HT), and further performed MR-based mediation analysis to ascertain the mediating role of of 731 immunophenotypes.

## 2 Materials and methods

### 2.1 Study design

**Figure 1:**
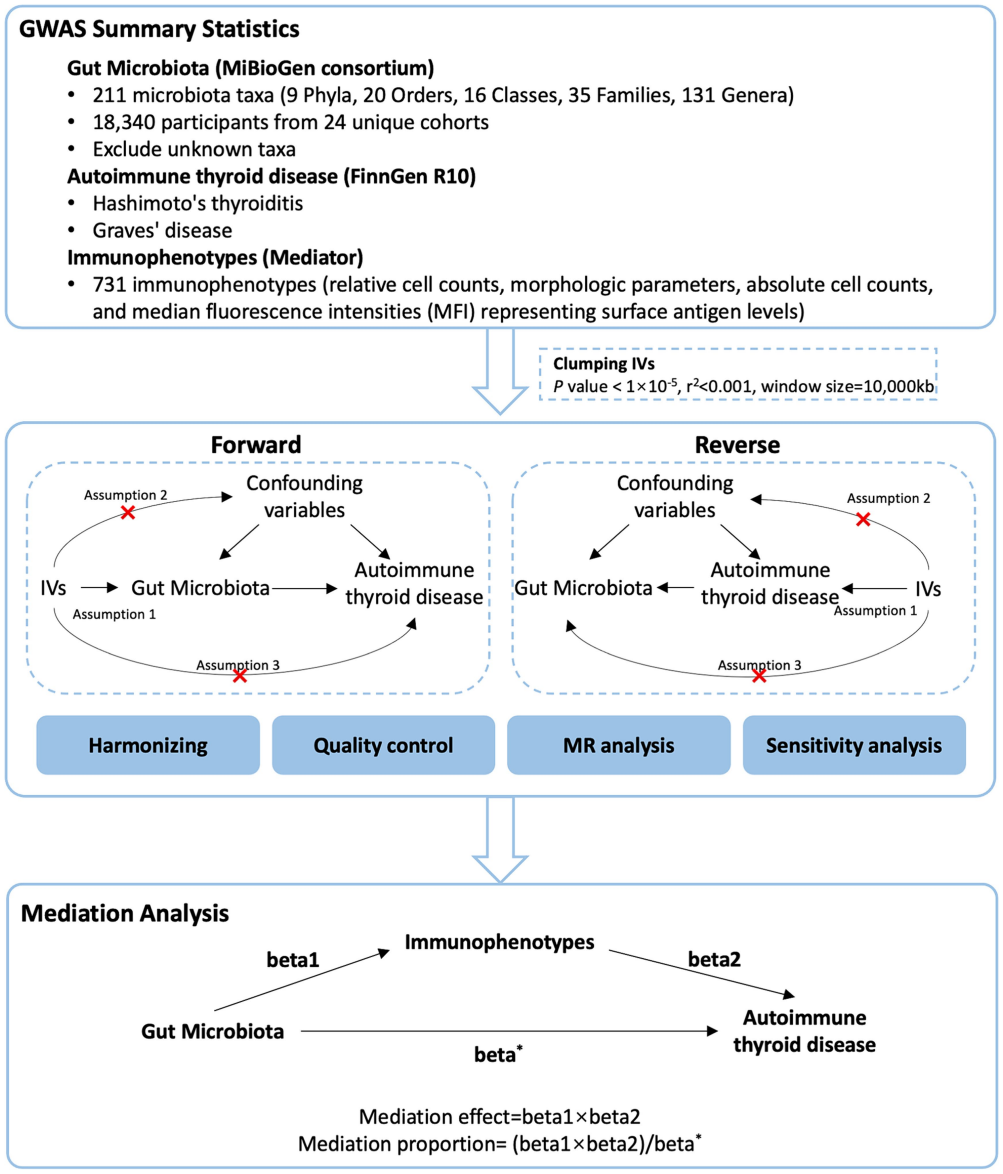
Study flow chart. The entire workflow of MR analysis.

We used a two-sample MR design: a genetic instrumental variable analysis based on summary-level data with SNPs as instruments for the risk factor. For causal estimates from MR studies to be valid, three assumptions must be met (Sekula et al., 2016): (1) the genetic variants are strongly associated with the exposure, (2) the genetic variants are not associated with any potential confounder of the exposure-outcome association and (3) the variants do not affect outcome independently of exposure. We first performed a two-sample bidirectional MR to assess the association of GM with AITD. Then, we applied a two-step MR analysis to assess whether immune cells have a causal role in the mediating pathway between GM and AITD. This study is reported following the Strengthening the Reporting of Observational Studies in Epidemiology Using Mendelian Randomization guidelines (STROBE-MR, S1 Checklist) (Skrivankova et al., 2021). The study’s design and progression are illustrated in Figure 1.

### 2.2 Data source

The summary statistics of GM were retrieved from the MiBioGen consortium,^1^ which serves as a vast database, diligently compiling and analyzing genome-wide genotypes alongside 16S fecal microbiome data. This dataset includes 18,340 participants from 24 unique cohorts, and the GWAS summary data included a total of 211 GM taxa (131 genera, 35 families, 20 orders, 16 classes, and 9 phyla). The GWAS summary data of HT and GD were derived from the Finngen R10 consortium. A public catalog (GCST0001391 to GCST0002121) containing GWAS data for 731 immunophenotypes was included in the study. A total of 731 immunophenotypes were examined, including relative cell counts (RC) (192), morphologic parameters (MP) (32), absolute cell counts (AC) (118), and median fluorescence intensities (MFI) representing surface antigen levels (389). Of these, MP features included CDC and TBNK panels, whereas MFI, RC, and AC features included B cells, CDC, T cell maturation stage, myeloid cells, monocytes, and TBNK (T cells, B cells, natural killer proteins).

The present study is a secondary analysis of publicly available GWAS summary statistics. Ethical approval was granted for each of the original GWAS studies. In addition, no individual-level data was used in this study. Therefore, no new ethical review board approval was required.

### 2.3 IVs selection

The following selection criteria were used to choose the IVs: (1) SNPs associated with each genus at the locus-wide significance threshold (*p* < 1.0 × 10^5^) were selected as potential IVs; (2) 1000 Genomes project European samples data were used as the reference panel to calculate the linkage disequilibrium (LD) between the SNPs, and among those SNPs that had R^2^ < 0.001 (clumping window size = 10,000 kb), only the SNPs with the lowest *p* values were retained; (3) SNPs with minor allele frequency (MAF) ≤ 0.01 were removed; and (4) when palindromic SNPs existed, the forward strand alleles were inferred using allele frequency information.

### 2.4 Statistical analysis

We conducted a bidirectional two-sample MR analysis to assess the connection between GM and AITD. Our main analysis employed the inverse variance-weighted (IVW) meta-analysis method. To enhance the reliability of our findings, we also performed additional analyses using the weighted median, MR-Egger regression, simple mode and weighted mode methods. We evaluated the potential influence of directional pleiotropy by examining the intercept value in the MR-Egger regression. MR-Egger intercept *p*-value exceeding 0.05 indicated the absence of pleiotropy. The MR PRESSO was utilized to detect pleiotropy and outliers. We gauged heterogeneity using Cochran’s Q test. When faced with heterogeneity, we chose a random-effects IVW for our primary analysis.

The mediation analysis by a multivariable Mendelian randomization (MVMR) approach in this study focused on AITD-related GM and immunophenotypes. In addition to the basic effect estimates of GM on AITD (beta*) obtained from the univariate MR analyses, two more estimates were calculated: (1) the effect of the immunophenotypes on AITD adjusting for bacteria (beta2), and (2) the causal effect of the exposure (significant GM on AITD in primary MR analysis) on the mediator (AITD-related immunophenotypes) (beta1). The mediation effect, which refers to the causal effect of GM on AITD via mediators, can then be calculated by using the following formula: beta1 × beta2. Thus, mediation proportion could be calculated as “indirect effect/total effect” ([beta1 × beta2]/beta*).

All statistical analyses were conducted using the R software, version 4.3.1. MR analyses were performed using the TwoSampleMR (version 0.5.6) and MR-PRESSO (version 1.0) R packages.

## 3 Results

### 3.1 Genetic causality and correlation between GM and AITD

The number of SNPs identified as IVs ranged from 3 to 26 (median: 12) for the 211 GM taxa, and the median F-statistic was 21.03 (ranged from 14.58 to 88.42) (Supplementary Tables S1, S2).

**Figure 2:**
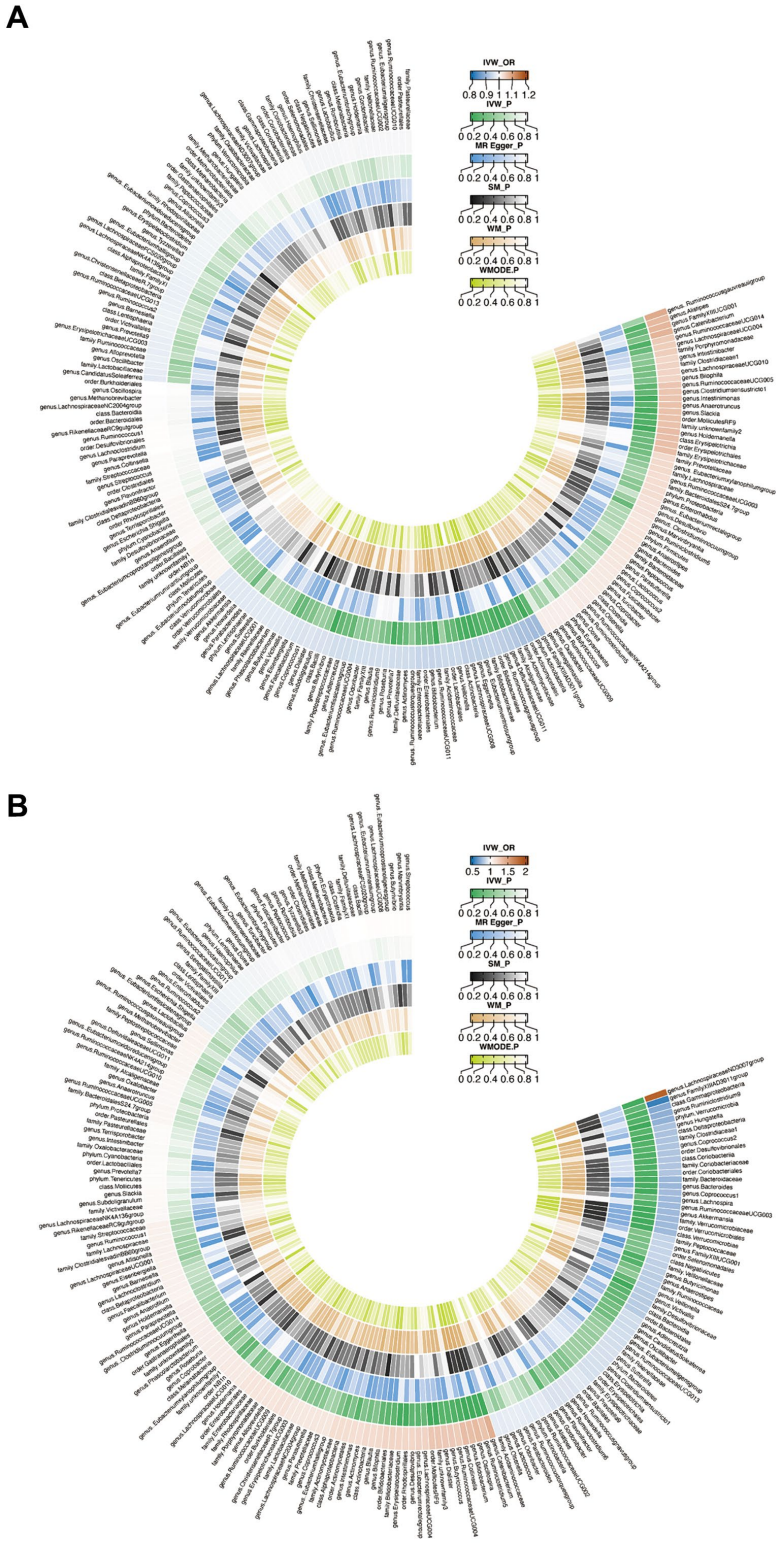
Circus plot of Mendelian randomization analyses of gut microbiota (GM) on autoimmune thyroid disease. **(A)** Mendelian randomization analysis between GM and Hashimoto’s thyroiditis. **(B)** Mendelian randomization analysis between GM and Graves’ disease. IVW_OR, the results of odds ratio of inverse variance weighted method. IVW_P, the *p* value for inverse variance weighted method. MR Egger_P, the p value for MR Egger method. SM_P, the *p* value for Simple mode method. WM_P, the p value for weighted median method. WMODE.P, the *p* value for weighted mode method.

**Figure 3:**
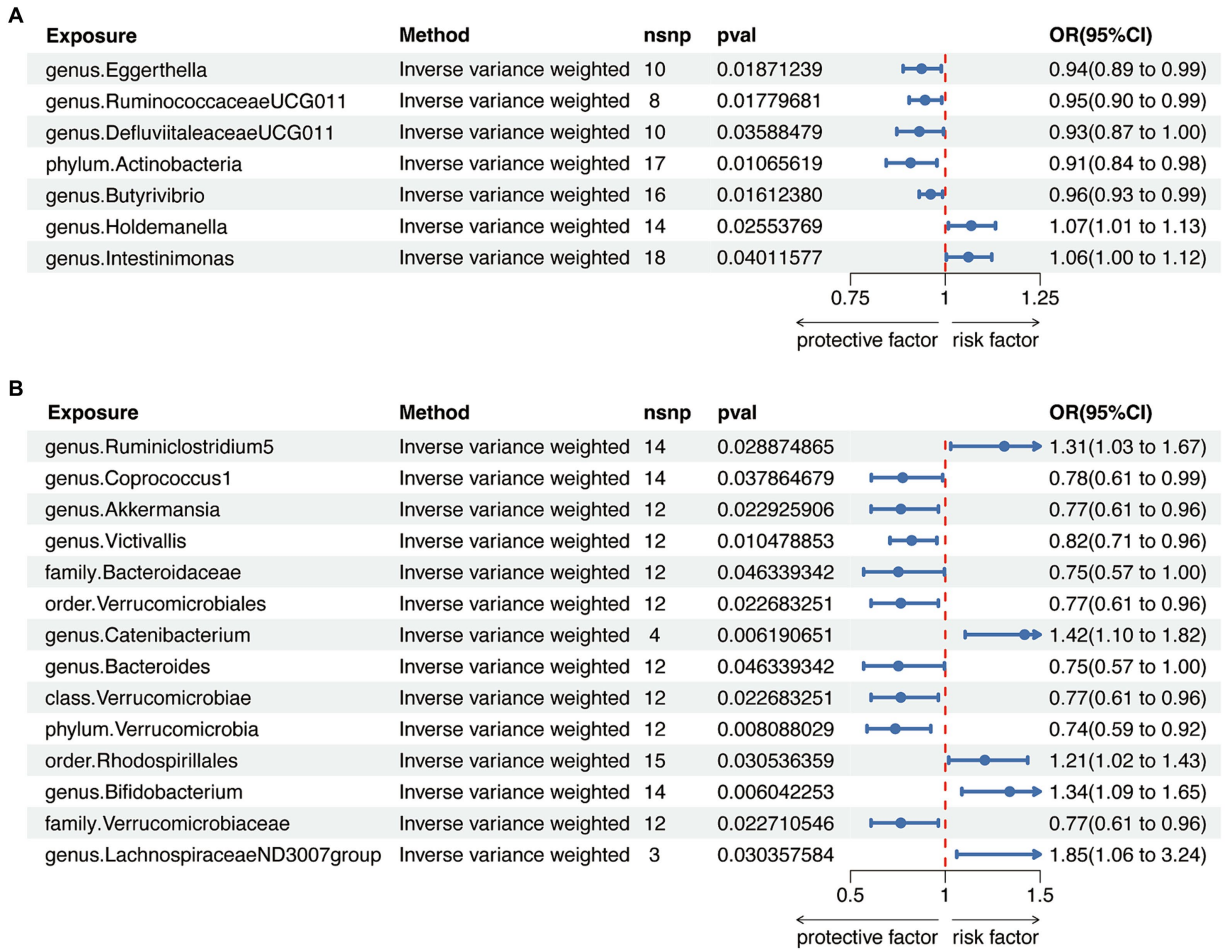
Mendelian randomization analysis between gut microbiota (GM) and autoimmune thyroid disease. **(A)** Mendelian randomization analysis between GM and Hashimoto’s thyroiditis. **(B)** Mendelian randomization analysis between GM and Graves’ disease.

When evaluating the causal effects of GM on HT, six genera and one phylum were significantly associated with HT (Figures 2A, 3A, Supplementary Table S3). The abundance of *Eggerthella (g)* (IVW: OR 0.94, 95% CI 0.89–0.99, *p* = 0.019), *RuminococcaceaeUCG011 (g)* (IVW: OR 0.95, 95% CI 0.90–0.99, *p* = 0.018), *DefluviitaleaceaeUCG011 (g)* (IVW: OR 0.93, 95% CI 0.87–1.00, *p* = 0.036), *Actinobacteria (p)* (IVW: OR 0.91, 95% CI 0.84–0.98, *p* = 0.011), and *Butyrivibrio (g)* (IVW: OR 0.96, 95% CI 0.93–0.99, *p* = 0.016) presented protective effect on HT. Conversely, the abundance of *Holdemanella (g)* (IVW: OR 1.07, 95% CI 1.01–1.13, *p* = 0.026) and *Intestinimonas (g)* (IVW: OR 1.06, 95% CI 1.00–1.12, *p* = 0.040) increased the risk of HT. No heterogeneity and pleiotropy were found in (Supplementary ables S4, S5) SNPs.

When considering GD as the outcome, eight genera, two families, two orders, one class and one phylum showed a significant correlation (Figures 2B, 3B, Supplementary Table S6). *Coprococcus1 (g)* (IVW: OR 0.78, 95% CI 0.61–0.99, *p* = 0.037), *Akkermansia (g)* (IVW: OR 0.77, 95% CI 0.61–0.96, *p* = 0.023), *Victivallis (g)* (IVW: OR 0.82, 95% CI 0.71–0.96, *p* = 0.010), *Bacteroidaceae (f)* (IVW: OR 0.75, 95% CI 0.57–1.00, *p* = 0.046), *Verrucomicrobiales (o)* (IVW: OR 0.77, 95% CI 0.61–0.96, *p* = 0.023), *Bacteroides (g)* (IVW: OR 0.75, 95% CI 0.57–1.00, *p* = 0.046), *Verrucomicrobiae (c)* (IVW: OR 0.77, 95% CI 0.61–0.96, *p* = 0.023), *Verrucomicrobia (p)* (IVW: OR 0.74, 95% CI 0.59–0.92, *p* = 0.008) and Verrucomicrobiaceae (v) (IVW: OR 0.77, 95% CI 0.61–0.96, *p* = 0.023) had protective effect on GD, while the increased abundance of *Ruminiclostridium5 (g)* (IVW: OR 1.31, 95% CI 1.03–1.67, *p* = 0.029), *Catenibacterium (g)* (IVW: OR 1.42, 95% CI 1.10–1.82, *p* = 0.006), *Rhodospirillales (o)* (IVW: OR 1.21, 95% CI 1.02–1.43, *p* = 0.031), *Bifidobacterium (g)* (IVW: OR 1.34, 95% CI 1.09–1.65, *p* = 0.006) and *LachnospiraceaeND3007group (g)* (IVW: OR 1.85, 95% CI 1.06–3.24, *p* = 0.030) were positively associated with the risk of GD. The above results also demonstrated an absence of heterogeneity and pleiotropy (Supplementary Tables S7, S8).

**Figure 4:**
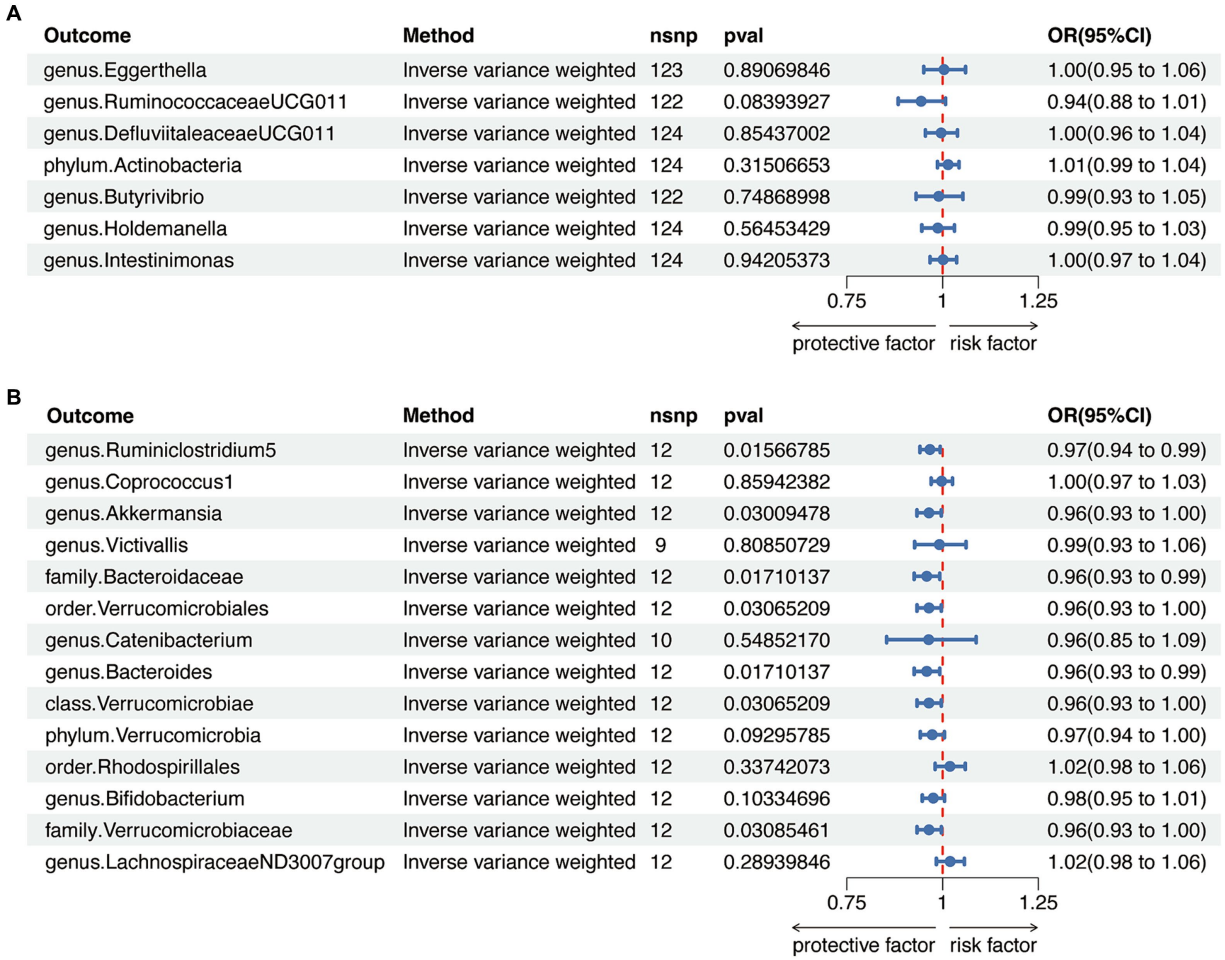
Reverse Mendelian randomization analysis between gut microbiota (GM) and autoimmune thyroid disease. **(A)** Reverse Mendelian randomization analysis between GM and Hashimoto’s thyroiditis. **(B)** Reverse Mendelian randomization analysis between GM and Graves’ disease.

To avoid bidirectional effects, we further valuated the causal effects of AITD on above-mentioned GM taxa (Supplementary Tables S9, S10). All included taxa did not exhibit significant variation after HT, while the relative abundance of one class, one order, two families, and three genera were significantly altered after GD (Figure 4 and Supplementary Tables S11, S12). Therefore, after removing taxa with bidirectional effects, a total of six genera and one phylum were included for HT, and five genera, one order and one phylum were included for GD in further analysis.

### 3.2 Mediator screening

Aiming to identify potential mediators, we utilized 731 immune cell traits to investigate their effects on AITD. The number of SNPs identified as IVs ranged from 3 to 713 (median: 22) for the 731 immunophenotypes, and the median F-statistic was 21.66 (ranged from 9.54 to 2380.38) (Supplementary Tables S13, S14).

**Figure 5:**
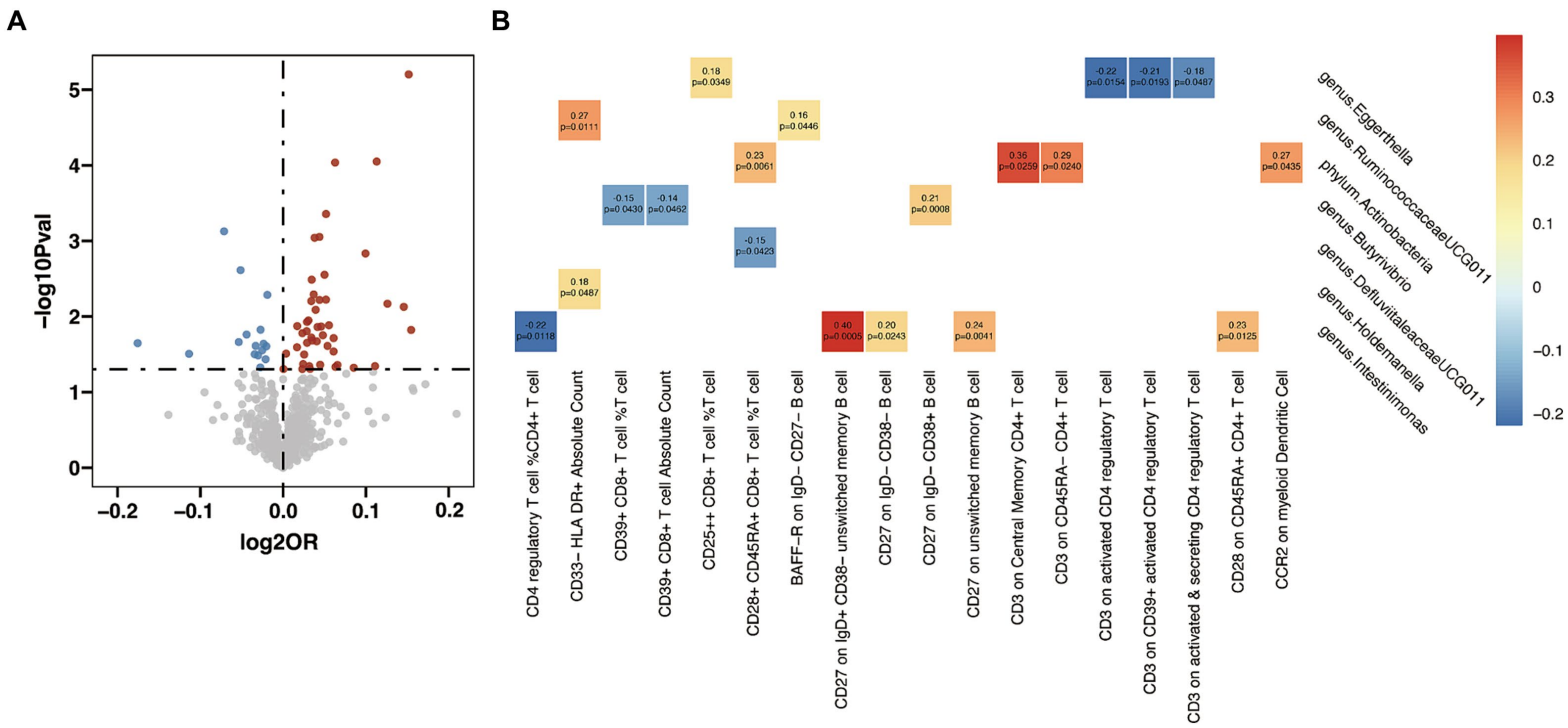
Mendelian randomization analysis between gut microbiota (GM) and mediator in Hashimoto’s thyroiditis (HT). **(A)** Volcano plot of immunophenotypes with significant association with HT. **(B)** Mendelian randomization analysis between GM and immunophenotypes.

In the analysis examining the association between immune cell traits and HT, we found 46 immunophenotypes were positively correlated with HT and 16 were negatively correlated (Figure 5A and Supplementary Table S15). Furthermore, we explored the potential mediation effects of GM exposures on these significant mediators in HT (Figure 5B). Significant mediation effects were found in six GM taxa. *Eggerthella (g)* showed various mediation effect on HT via four different mediators: CD25++ CD8+ T cell %T cell (*β* = 0.18, *p* = 0.035), CD3 on activated CD4 regulatory T cell (*β* = −0.22, *p* = 0.015), CD3 on CD39+ activated CD4 regulatory T cell (*β* = −0.21, *p* = 0.019), and CD3 on activated & secreting CD4 regulatory T cell (*β* = −0.18, *p* = 0.049). *RuminococcaceaeUCG011 (g)* demonstrated a positive mediation effect through CD33-HLA DR+ Absolute Count (*β* = 0.27, *p* = 0.011) and BAFF-R on ID- CD27- B cell (*β* = 0.16, *p* = 0.045). *Actinobacteria (p)* showed positive effect on HT via four different mediators: CD28+ CD45RA+ CD8+ T cell %T cell (*β* = 0.23, *p* = 0.006), CD3 on Central Memory CD4+ T cell (*β* = 0.36, *p* = 0.026), CD3 on CD45RA- CD4+ T cell (*β* = 0.29, *p* = 0.024), and CCR2 on myeloid Dendritic Cell (*β* = 0.27, *p* = 0.044). *Butyrivibrio (g)* presented mixed mediation effect through CD39+ CD8+ T cell %T cell (*β* = −0.15, *p* = 0.043), CD39+ CD8+ T cell Absolute Count (*β* = −0.14, *p* = 0.046) and CD27 on lgD- CD38+ B cell (*β* = 0.21, *p* = 0.001). *Holdemanella (g)* also showed mixed mediation effect, via CD33-HLA DR+ Absolute Count (*β* = 0.18, *p* = 0.049) and CD28+ CD45RA+ CD8+ T cell %T cell (*β* = −0.15, *p* = 0.042), respectively. Strongest mediation effect was found in *Intestinimonas (g)* though CD27 on ID+ CD38- unswitched memory B cell (*β* = 0.40, *p* = 0.001), as well as CD27 on lgD- CD38- B cell (*β* = 0.20, *p* = 0.024), CD27 on unswitched memory B cell (*β* = 0.24, *p* = 0.004), CD28 on CD45RA+ CD4+ T cell (*β* = 0.23, *p* = 0.013) and CD4 regulatory T cell %CD4+ T cell (*β* = −0.22, *p* = 0.012).

**Figure 6:**
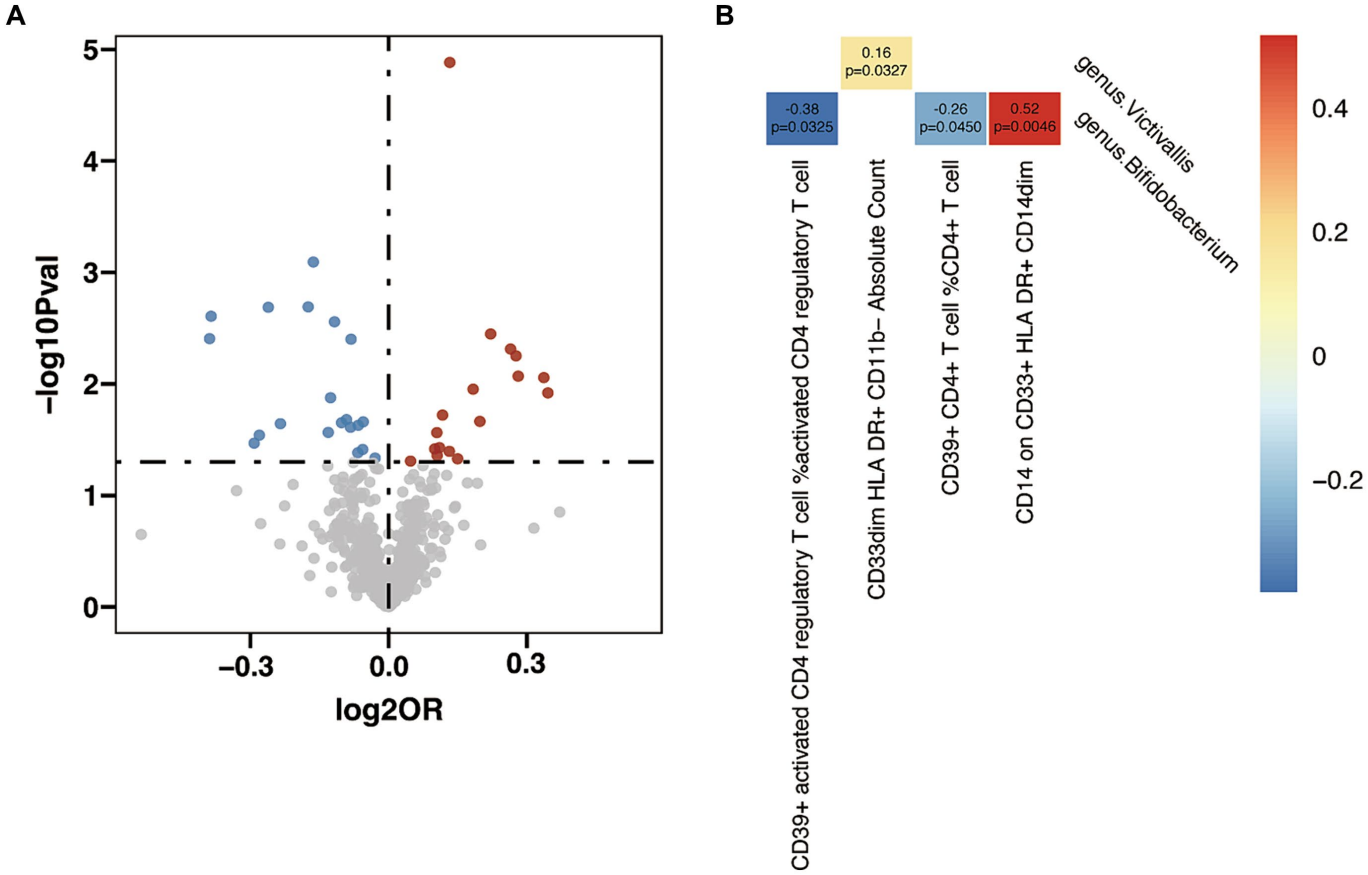
Mendelian randomization analysis between gut microbiota (GM) and mediator in Graves’ disease (GD). **(A)** Volcano plot of immunophenotypes with significant association with GD. **(B)** Mendelian randomization analysis between GM and immunophenotypes.

In the analysis between immune cell traits and GD, 17 immunophenotypes showed a positive correlation with GD and 20 showed a negative correlation (Figure 6A and Supplementary Table S16). Investigation of the potential mediation effects of GM exposures on these mediators in GD unveiled two GM genera (Figure 6B). *Victivallis (g)* presented positive effect on GD via CD33dim HLA DR+ CD11b- Absolute Count (*β* = 0.16, *p* = 0.033), and *Bifidobacterium (g)* showed mixed effect though three mediators: CD39+ activated CD4 regulatory T cell %activated CD4 regulatory T cell (*β* = −0.38, *p* = 0.033), CD39+ CD4+ T cell %CD4+ T cell (*β* = −0.26, *p* = 0.045) and CD14 on CD33+ HLA DR+ CD14dim (*β* = 0.52, *p* = 0.005).

### 3.3 Mediation analyses of potential immunophenotypes

After pinpointing significant mediators influencing AITD and the subsequent effects of exposure on mediation, we quantified the mediation effect proportions (Table 1). As for HT, *Intestinimonas (g)* exhibited mediation effects via CD4 regulatory T cell %CD4+ T cell (11.2%), CD27 on IgD+ CD38- unswitched memory B cell (10.8%), CD27 on IgD- CD38- B cell (11.9%), CD27 on unswitched memory B cell (11.1%) and CD28 on CD45RA+ CD4+ T cell (30.2%). *Eggerthella (g)* mediated its effects on HT through CD3 on activated CD4 regulatory T cell (8.8%), CD3 on CD39+ activated CD4 regulatory T cell (7.9%) and CD3 on activated & secreting CD4 regulatory T cell (7.4%). Other notable mediation effects included *Holdemanella (g)* via CD33- HLA DR+ Absolute Count (3.3%), *DefluviitaleaceaeUCG011 (g)* via CD28+ CD45RA+ CD8+ T cell %T cell (0.5%), *Actinobacteria (p)* via CCR2 on myeloid Dendritic Cell (5.0%) and *RuminococcaceaeUCG011 (g)* via BAFF-R on IgD- CD27- B cell (4.4%). When considering GD, *Bifidobacterium (g)* showed its mediation effects through CD39+ CD4+ T cell %CD4+ T cell (5.0%) and CD14 on CD33+ HLA DR+ CD14dim (12.2%), and *Victivallis (g)* showed effects on GD through CD33dim HLA DR+ CD11b- Absolute Count (3.3%).

**Table 1:** Mediation analysis of immunophenotypes between gut microbiota and autoimmune thyroid disease.

Exposure	Mediator	Outcome	Total effect	Direct effect	Mediation effect (95% CI)	*P* value	Mediation proportion (95% CI)
*g_Intestinimonas*	CD4 regulatory T cell %CD4+ T cell	Hashimoto’s thyroiditis	0.0591	0.0525	0.0066 (−0.0302,0.0435)	0.7249	11.2% (−51.1,73.5%)
*g_Intestinimonas*	CD27 on IgD+ CD38- unswitched memory B cell	Hashimoto’s thyroiditis	0.0591	0.0527	0.0064 (−0.0825, 0.0952)	0.8882	10.8% (−140, 161%)
*g_Intestinimonas*	CD27 on IgD- CD38- B cell	Hashimoto’s thyroiditis	0.0591	0.0521	0.0071 (−0.0270, 0.0411)	0.6849	11.9% (−45.7, 69.6%)
*g_Intestinimonas*	CD27 on unswitched memory B cell	Hashimoto’s thyroiditis	0.0591	0.0525	0.0066 (−0.0334, 0.0466)	0.7472	11.1% (−56.5, 78.8%)
*g_Intestinimonas*	CD28 on CD45RA+ CD4+ T cell	Hashimoto’s thyroiditis	0.0591	0.0413	0.0179 (−0.0266, 0.0623)	0.4313	30.2% (−45.0, 105.0%)
*g_Holdemanella*	CD33- HLA DR+ Absolute Count	Hashimoto’s thyroiditis	0.0662	0.0640	0.0022 (−0.0313, 0.0357)	0.8991	3.3% (−47.3, 53.9%)
*g_DefluviitaleaceaeUCG011*	CD28+ CD45RA+ CD8+ T cell %T cell	Hashimoto’s thyroiditis	−0.0708	−0.0705	−0.0004 (−0.0231, 0.0224)	0.9740	0.5% (32.7, −31.6%)
*g_Butyrivibrio*	CD39+ CD8+ T cell %T cell	Hashimoto’s thyroiditis	−0.0393	−0.0428	0.0035 (−0.0178, 0.0248)	0.7471	−8.9% (45.3, −63.2%)
*g_Butyrivibrio*	CD39+ CD8+ T cell Absolute Count	Hashimoto’s thyroiditis	−0.0393	−0.0420	0.0027 (−0.0174, 0.0227)	0.7930	−6.8% (44.2, −57.9%)
*g_Butyrivibrio*	CD27 on IgD- CD38+ B cell	Hashimoto’s thyroiditis	−0.0393	−0.0536	0.0143 (−0.0121, 0.0407)	0.2888	−36.3% (30.8, −104%)
*p_Actinobacteria*	CD28+ CD45RA+ CD8+ T cell %T cell	Hashimoto’s thyroiditis	−0.0959	−0.0965	0.0006 (−0.0380, 0.0391)	0.9767	−0.6% (39.6, −40.8%)
*p_Actinobacteria*	CD3 on Central Memory CD4+ T cell	Hashimoto’s thyroiditis	−0.0959	−0.1045	0.0086 (−0.1070, 0.1240)	0.8846	−8.9% (111, −129%)
*p_Actinobacteria*	CD3 on CD45RA- CD4+ T cell	Hashimoto’s thyroiditis	−0.0959	−0.1045	0.0086 (−0.0694, 0.0866)	0.8284	−9.0% (72.3, −90.3%)
*p_Actinobacteria*	CCR2 on myeloid Dendritic Cell	Hashimoto’s thyroiditis	−0.0959	−0.0911	−0.0048 (−0.0771, 0.0675)	0.8966	5% (80.4, −70.4%)
*g_RuminococcaceaeUCG011*	CD33- HLA DR+ Absolute Count	Hashimoto’s thyroiditis	−0.0548	−0.0580	0.0032 (−0.0536, 0.0600)	0.9120	−5.8% (97.8, −109%)
*g_RuminococcaceaeUCG011*	BAFF-R on IgD- CD27- B cell	Hashimoto’s thyroiditis	−0.0548	−0.0525	−0.0024 (−0.0285, 0.0237)	0.8578	4.4% (51.9, −43.2%)
*g_Eggerthella*	CD25++ CD8+ T cell %T cell	Hashimoto’s thyroiditis	−0.0644	−0.0696	0.0052 (−0.0269, 0.0373)	0.7503	−8.1% (41.7, −57.9%)
*g_Eggerthella*	CD3 on activated CD4 regulatory T cell	Hashimoto’s thyroiditis	−0.0644	−0.0588	−0.0056 (−0.0452, 0.0339)	0.7799	8.8% (70.2, −52.7%)
*g_Eggerthella*	CD3 on CD39+ activated CD4 regulatory T cell	Hashimoto’s thyroiditis	−0.0644	−0.0593	−0.0051 (−0.0426, 0.0325)	0.7920	7.9% (66.2, −50.5%)
*g_Eggerthella*	CD3 on activated & secreting CD4 regulatory T cell	Hashimoto’s thyroiditis	−0.0644	−0.0597	−0.0048 (−0.0372, 0.0277)	0.7747	7.4% (57.8, −43.1%)
*g_Bifidobacterium*	CD39+ activated CD4 regulatory T cell %activated CD4 regulatory T cell	Graves’ disease	0.2925	0.3320	−0.0396 (−0.1780, 0.0990)	0.5757	−13.5% (−60.9,33.8%)
*g_Bifidobacterium*	CD39+ CD4+ T cell %CD4+ T cell	Graves’ disease	0.2925	0.2779	0.0146 (−0.0515, 0.0806)	0.6659	5.0% (−17.6, 27.6%)
*g_Bifidobacterium*	CD14 on CD33+ HLA DR+ CD14dim	Graves’ disease	0.2925	0.2567	0.0358 (−0.1510, 0.2230)	0.7074	12.2% (−51.6, 76.1%)
*g_Victivallis*	CD33dim HLA DR+ CD11b- Absolute Count	Graves’ disease	−0.1953	−0.1890	−0.0063 (−0.0320, 0.0193)	0.6283	3.3% (16.4, −9.9%)

## 4 Discussion

The intricate relationship between the GM and immune-mediated diseases has been a topic of burgeoning interest in recent years. The present large-scale MR study delved into the associations between specific GM taxa and AITD, and analyzed the mediating role of immune cell traits in this complex interplay. A total of 7 GM taxa were found to be positively associated with AITD, suggesting that abundance of them might be linked to a higher risk of developing AITD. Other 14 taxa showed negative correlation with AITD, indicating a potential protective effect against the disease. Further analysis regarding mediation effects revealed that the above-mentioned GM taxa could affect the onset of AITD through diverse immune cell traits, providing novel insights into the GM-immune system-AITD regulating axis.

As mentioned in the introduction, the gut-thyroid communication hints at a possible influence of the GM on AITD onsets. Previous studies demonstrated that the gut could influence the thyroid function through several microbial-related mechanisms. Dysbiosis of microbiota leads to the damaged intestinal barrier and increased intestinal permeability, allowing the antigens to pass into the circulation and activate the immune system (Cayres et al., 2021). In addition, the antibodies in the circulation may react with the bacterial antigen and enhance the activation of the inflammasome in thyroid, which can be modulated by the GM and its metabolism in turn (Tomasello et al., 2015; Yao et al., 2017; Guo et al., 2018). Although many researchers have found that AITD patients have reduced α diversity and abundances of certain microbiota compared with healthy controls (Zhao et al., 2018; Liu et al., 2020), there is no direct evidence of the cause-effect relationship between AITD and GM. Our results revealed several GM taxa showing significant association with HT and GD. Among them, *Actinobacteria (p)* presented the most notable protective effects on HT. *Actinobacteria* is one the four major phyla of the GM and is pivotal in the maintenance of gut homeostasis (Binda et al., 2018; Byrd et al., 2021). Numerous *Actinobacteria* spp. participate in the maintenance of microbial homeostasis, with some being considered as probiotics that potentially exert a protective influence on hypothyroidism (Liu et al., 2023; Xie et al., 2023), which are in accordance with our result. Surprisingly, genus *Bifidobacterium*, as a probiotic strain within the phylum *Actinobacteria*, presented a significantly facilitating effects on GD. *Bifidobacterium* spp. is a widely used probiotic as it confers several physiological benefits to humans, and it was traditionally deemed as a protective genus against thyroid disease in previous studies (Butel, 2014). To be noted, *Bifidobacterium* spp. may be protective or progressive in autoimmune disorders, such as another MR study from Xu et al. revealed that a higher relative abundance of the *Bifidobacterium* spp. genus was associated with a higher risk of type 1 diabetes (Xu et al., 2021). Thus, their involvement in immunopathogenesis is still unclear and requires future mechanistic studies. It is worth mentioning that even though dysbiosis is often used to indicate the discrepancy in microbiota between patients and healthy group, the notion of “dysbiosis” is a broad term used lately as a mental shortcut. It should be kept in mind that GM is an intricate ecosystem and there is no accepted definition of “healthy” or “detrimental” microbiota as we discuss the complex effect of GM (Hooks and O'Malley, 2017; Mitrea et al., 2022).

In addition to the generation of thyroid autoantibodies and abnormal thyroid hormone production, AITD histologically involves the infiltration of self-targeting T and B lymphocytes in the thyroid gland. Thus, our analyses also provided genetic evidence for the involvement of immune cells in the causal correlation between GM and AITD. The GM largely regulates the homeostasis as well as the development of immune cells. It modulates both the innate and the adaptive immune system, even outside the gut (Maslowski and Mackay, 2011), and is fundamental in the development of gut-associated lymphatic tissue, where more than 70% of the entire immune system is situated (Virili et al., 2018). The microbiome and immune system share a complex relationship, and disruptions in this balance can lead to immune disorders (Zheng et al., 2020). Our mediation analysis find some immune cell trait can participate the effect of microbiota on AITD. The phylum *Actinobacteria* showed a mediating effect on HT via CCR2 on myeloid Dendritic Cell. Chemokine receptor 2 (CCR2) is the receptor of chemokine ligand 2 (CCL2) and is involved in recruiting monocytes and macrophages to sites of inflammation. In autoimmune diseases like rheumatoid arthritis, multiple sclerosis, and inflammatory bowel disease, CCR2 promotes the migration of inflammatory immune cells to the affected tissues, contributing to tissue damage and perpetuation of the immune response, but its role in AITD remains to be discovered. Results from the present study suggested that the phylum *Actinobacteria* influenced the onset of HT through the CCL2/CCR2 signaling axis, which might be worth future investigation. In the mediation analysis for GD, *Bifidobacterium* mediated its effects mainly through CD14 on CD33+ HLA DR+ CD14dim. CD14 is a glycoprotein receptor expressed on the surface of various immune cells, including monocytes, macrophages, and neutrophils, and it plays an important role in the innate immune response and is involved in the recognition and response to microbial pathogens (Wu et al., 2019). Jia et al. performed bioinformatics analysis and demonstrated the significant association between genetic variations in CD14 and GD, and further discovered that CD14 expression level was positively correlated with the proportion of macrophages M1 cell and M1/M2 ratio in GD thyroid tissues (Jia et al., 2018). CD14 may contribute to the recognition and presentation of self-antigens in GD, promoting the activation of immune responses against the thyroid tissue, but the specific involvement of CD14 in thyroid disease is still an area of ongoing research.

There are some drawbacks in the present study. First of all, the majority of patients in the GWAS summary data used in our study were European, which might introduce certain biases and restrict the broader applicability of our findings to other ethnic groups. Besides, the characterization of microbiome profiles in the MiBioGen consortium only allows resolution from the genus to phylum, which might result in lacking details on the species level. Furthermore, MR assumes a linear relationship between exposure and outcome, but the relationship may be more complex, involving nonlinear relationships and interactions with other environmental and genetic factors. Overall, future randomized controlled trials of AITD would be the next step in this study in order to reduce the potential impact of confounding factors and thus obtain a higher level of evidence for causality.

## 5 Conclusion

In summary, we comprehensively assessed the causal association between the GM and AITD and further analyzed the mediation effects of multiple immune cell traits. We demonstrated significant effects of phylum *Actinobacteria* on HT and genus *Bifidobacterium* on GD. The identified associations and mediation effects pave the way for future research, emphasizing the importance of the gut-immune axis in disease onset. Potential therapeutic interventions targeting the GM could be explored as novel strategies for managing AITD.

## Data Availability

The original contributions presented in the study are included in the article/Supplementary material, further inquiries can be directed to the corresponding authors.

## Footnotes

^1^ https://mibiogen.gcc.rug.nl/
